# Anterior versus posterior approach in Lenke 5C adolescent idiopathic scoliosis: a meta-analysis of fusion segments and radiological outcomes

**DOI:** 10.1186/s13018-016-0415-9

**Published:** 2016-07-11

**Authors:** Ming Luo, Wengang Wang, Mingkui Shen, Lei Xia

**Affiliations:** Institute of Spinal Deformity, The First Affiliated Hospital of Zhengzhou University, Zhengzhou, Henan 450000 People’s Republic of China

**Keywords:** Lenke 5C, Adolescent idiopathic scoliosis, Anterior approach, Posterior approach, Meta-analysis

## Abstract

**Background:**

Radiological outcomes between anterior and posterior approach in Lenke 5C curves were still controversial. Meta-analysis on published articles to compare fusion segments and radiological outcomes between the two surgical approaches was performed.

**Methods:**

Electronic database was conducted for searching studies concerning the anterior versus posterior approach in Lenke 5C curves. After quality assessment, data of means, standard deviations, and sample sizes were extracted. RevMan 5.3 was adopted for data analysis.

**Results:**

Seven case-control studies involving 308 Lenke 5C AIS patients were identified in the meta-analysis. No significant differences were noted in correction rate of thoracolumbar/lumbar curve (95 % CI −6.02 to 4.32, *P* = 0.75) and incidence of proximal junctional kyphosis (95 % CI 0.12 to 7.19, *P* = 0.94) of final follow-up, in change values of thoracolumbar/lumbar curve (95 % CI −3.28 to 7.19, *P* = 0.46) and thoracic kyphosis (95 % CI −4.10 to 0.13, *P* = 0.07). The anterior approach represented a significant shorter fusion segments compared to posterior approach (95 % CI −1.72 to −0.71, *P <* 0.00001). The posterior approach obtained a larger increasing Cobb angle of lumbar lordosis than the anterior approach (95 % CI −6.06 to −0.61, *P* = 0.02).

**Conclusions:**

The anterior and posterior approach can obtain comparable coronal correction, change values of thoracic kyphosis, and incidence of proximal junctional kyphosis. The anterior approach saves approximate one more fusion segment, and the posterior approach can obtain a larger increasing Cobb angle of lumbar lordosis, from preoperation to final follow-up.

**Trial registration:**

The article type of this study is meta-analysis and prospective registration is not required.

## Background

Adolescent idiopathic scoliosis (AIS) afflicted 1–3 % adolescents at danger age of 10–16 years, and the pathogenesis continued to be obscure [1]. Lenke 5 AIS could be defined as a structural thoracolumbar/lumbar (TL/L) curve, with the upper thoracic and main thoracic curves were nonstructural. Lenke 5 AIS was then subdivided into A, B, or C according to lumbar spine modifier, and “C” indicates the central sacral vertical line medial to the lumbar apex [2]. The generally approved threshold for surgical treatment in AIS was a prime curve larger than 45° [1].

Anterior invasive technique was introduced by Dwyer and Schafer [3]. From the primitive Dwyer cable to the later Zeilke-instrumentation, vertebral screw [4], and screw-single rod system, anterior approach had been practiced excellently in the correction of coronal plane [5]. With shorter fusion levels, anterior invasive technique was once the primary option for Lenke 5C curves. However, the disadvantages of the anterior approach were the poor derotation, kyphosis tendency, impairment of pulmonary function, and higher occurrence rate of implant breakage [6–8].

Posterior instrumentation continued to be the domination of surgical treatment for AIS patients*.* In 1962, Harrington initially introduced the operational invasive technique for spine deformity, which was regarded as the revolution of orthopedic surgery [9]. Cotrel-Dubousset system, the modern third-generation instrumentation, was constituted of segmental lamina grapple hooks and cross-linked double rods [10]. With the fantastic pull-out strength of pedicle screws comparing to traditional hooks, the pedicle screws resulted in the evolution of fourth-generation instrumentation [11–13]. However, the potential risk of implant malposition, neurological injury, increased operation time, and implant cost, which were related to pedicle screw placement, should be taken into a serious consideration [14–17].

Radiological outcomes between the two approaches in Lenke 5C curves were still controversial. Scholars used to be focused on the coronal correction and obtained different results [5, 13], and the sagittal correction had drawn more and more attentions in recent few years, especially for thoracic kyphosis (TK) and proximal junctional kyphosis (PJK; Cobb angle between the most proximal instrumented vertebra and the segment two levels cephalad) [18, 19]. In addition, to achieve a more stable correction with less fusion segments was the aim of each surgeon. The purpose of the current article was to compare the fusion segments, correction rate of TL/L curve, incidence of PJK, and change values of TL/L curve, TK, lumbar lordosis (LL) from preparation to final follow-up.

## Methods

### Search strategy

The searched database included the following: MEDLINE, the Web of Science, EMBASE, and Cochrane Library. Keywords included “Lenke 5” OR “thracolumbar/lumbar scoliosis”, and the publication date was from January 2005 to March 2016. In order to avoid potentially relevant studies escaped by the initial search, the “relevant items” and “references” of the included studies were also searched. In total of 717 potentially relevant studies identified from electronic databases. Two authors independently searched and extracted the data. Any difference was settled by mutual agreement.

### Selection criteria

Selection criteria: (1) AIS diagnosis; (2) Lenke 5C type; (3) case-control studies included anterior and posterior approach; (4) 1-year minimum follow-up; (5) articles published after January 2005; and (6) adequate data (sample size, mean, and SD) was provided for meta-analysis. All studies that did not fulfill the above principles were eliminated. In order to elucidate the possible repetition of patients, articles published in the space of a couple of years with similar title and authors were excluded.

### Data extraction

For the published studies that fulfill our inclusion criteria, information was cautiously extracted and computerized. The extracted variables were as follows (1) fusion segments; (2) Cobb angle of TL/L curve, TK, and LL, including preoperation and final follow-up; (3) correction rate of TL/L curve of final follow-up; (4) incidence of PJK of final follow-up; (5) study characteristics; (6) duration of follow-up; (7) sample size and gender of patients; (8) Risser sign; and (9) authors’ names and publication of year.

### Quality assessment

In order to reduce the risk of bias, the Cochrane Handbooks version 5.1.0 recommended the Newcastle-Ottawa scale (NOS) to evaluate the non-randomized controlled trial (RCT) articles. Rather than case reports, all the potentially relevant articles were case-control studies, so the NOS was competent for quality assessment. The NOS covers three dimensions to assess the included articles, including selection, comparability, and exposure [20]. Study with a score less than six was regarded as a high risk of bias, and it should be excluded. Quality assessment was undertaken by two independent reviews and differences being resolved by consensus if necessary.

### Statistical analysis

For statistical analysis, the RevMan software (the Cochrane Collaboration, Version 5.3) was adopted. With regard to continuous variables, such as correction rate of TL/L curve, mean different and 95 % confidence interval were presented, while dichotomous variables as incidence of PJK, odds ratios, and 95 % confidence interval were reported. When heterogeneity test showed *I*^2^ ≥ 50 %, we adopted the random effects model, and the fixed effect model was used when *I*^2^ < 50 %. In some studies, change values were not reported, a statistical transformation was conducted according to the data of preoperation and final follow-up, and the transformation formula we adopted was recommended by the Cochrane Handbooks version 5.1.0.

## Results

### Description of study

**Fig. 1:**
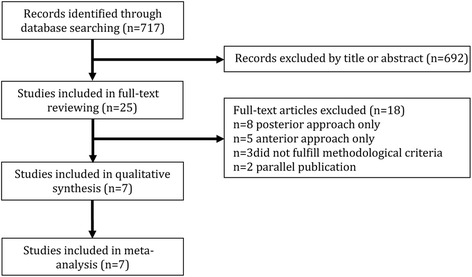
Flow diagram of study selection

Meta-analysis was performed on outcomes of seven case-control studies [21–27], two articles were excluded for parallel publication [28, 29]. The details of the study selection were shown in Fig. 1. All of the seven studies, 308 patients diagnosed with Lenke 5C AIS were included, and no significant differences were observed in age and preoperative TL/L Cobb angle between the anterior and posterior approach. The description information about the included articles was shown in Table 1.

**Table 1:** The description information about included articles

Study ID	Study type	Group	Number	Age, year	Gender (female/male)	Riser sign (range)	Follow-up, year	TL/L curves flexibility (%)	No. segments in lumbar curve	TL/L Cobb angle of preoperation	TL/L Cobb angle of final follow-up	Correction rate of TL/L Cobb angle of final follow-up
Hee et al. [21]	Retro	A	25	14.2 ± 1.5	25/0	0–5	≥2	NR	4.7 ± 0.7	50 ± 12	16 ± 9	NR
		P	11	14.5 ± 1.1	11/0	1–5	≥2	NR	4.8 ± 1.0	46 ± 5	15 ± 9	NR
Wang et al. [22]	Prosp	A	16	15.38 ± 1.54	15/1	2–5	≥2	79 ± 11	5 (4, 7)	42.56 ± 7.04	9.75 ± 4.12	77 ± 11
		P	16	14.88 ± 1.63	15/1	2–5	≥2	78 ± 17	5 (4, 7)	42.75 ± 6.12	7.56 ± 4.21	82 ± 10
Li et al. [23]	Retro	A	22	13.73 ± 1.32	NR	3 (2, 4)	≥2	47.05 ± 13.48	5 (5, 6)	50.18 ± 7.52	NR	54.42 ± 5.40
		P	24	13.58 ± 1.50	NR	2 (2, 3)	≥2	51.07 ± 10.27	5 (5, 7)	52.12 ± 6.40	NR	55.21 ± 6.05
Geck et al. [24]	Retro	A	31	15.6 ± 2.3	NR	NR	≥2	58.5	5.2 ± 0.74	49.0 ± 6.6	15.9 ± 9	66.6
		P	31	15.5 ± 2.0	NR	NR	≥2	50.3	5.7 ± 0.76	50.3 ± 7.0	8.0 ± 3.1	84.2
Zhan et al. [25]	Retro	A	22	14.5 ± 2.9	22/0	NR	≥1	NR	NR	56.0 ± 15.5	4.1 ± 2.0	93 ± 5
		P	20	14.8 ± 2.2	20/0	NR	≥1	NR	NR	53.0 ± 13.4	9.4 ± 6.3	88 ± 5
Eljure et al. [26]	Retro	A	18	15 ± 1.4	18/0	NR	≥2	NR	NR	NR	NR	NR
		P	19	15 ± 1.1	19/0	NR	≥2	NR	NR	NR	NR	NR
Dong et al. [27]	Retro	A	17	14.8 ± 1.8	14/3	NR	4.0 ± 2.2	87.4 ± 22.7	NR	41.4 ± 5.9	10.4 ± 8.4	75.4 ± 18.5
		P	36	14.5 ± 2.1	33/3	NR	4.0 ± 1.9	87.2 ± 27.5	NR	44.3 ± 7.4	7.5 ± 6.9	83.2 ± 16.3

*Retro* retrospective study; *Prosp* prospective study; *A* anterior approach; *P* posterior approach; *TL/L* thoracolumbar/lumbar

### Quality assessment

Seven articles were rated by two independent reviews, and differences were resolved by consensus if necessary. The score of the seven articles were six to eight according to the NOS, which suggested that all the seven articles should be included. The detailed quality assessment was showed in Table 2.

**Table 2:** Quality assessment according to the NOS

References	Selection	Comparability	Exposure	Total
Hee et al. [21]	3	2	2	7
Wang et al. [22]	3	2	3	8
Li et al. [23]	3	2	2	7
Geck et al. [24]	3	2	2	7
Zhan et al. [25]	2	2	2	6
Eljure et al. [26]	2	2	2	6
Dong et al. [27]	3	2	2	7

### Fusion segments

**Fig. 2:**
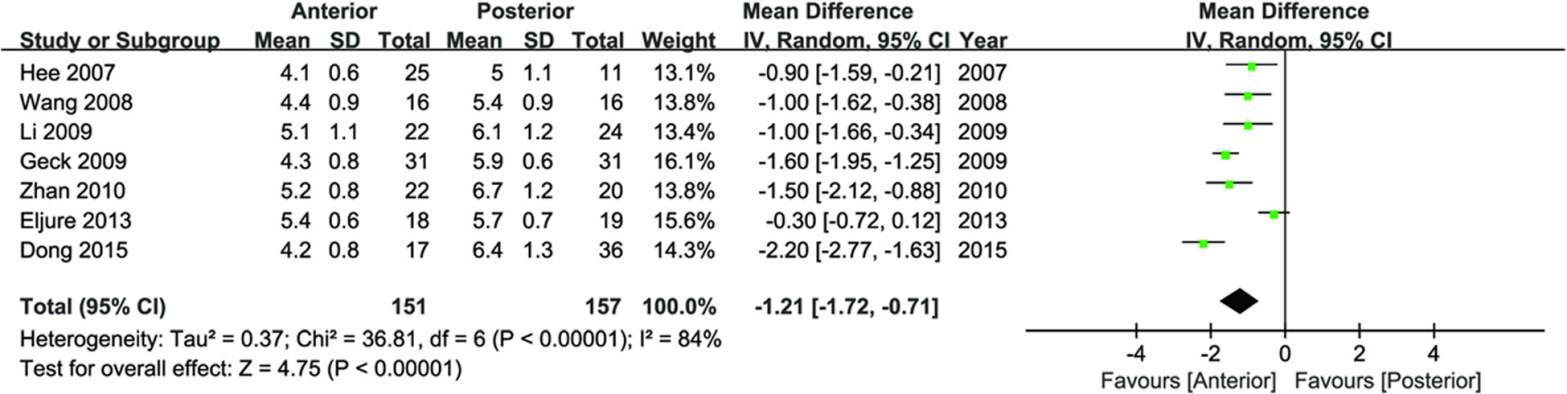
Forest plot for fusion segments

Data of the fusion segments were extracted from seven studies [21–27]. Compared with the posterior approach, anterior approach represented a significant shorter fusion segments (95 % CI −1.72 to −0.71, *P* < 0.00001; *I*^2^ = 84 %; Fig. 2), which suggested that the anterior approach saved approximate one fusion segment.

### Change values of TL/L curve

**Fig. 3:**
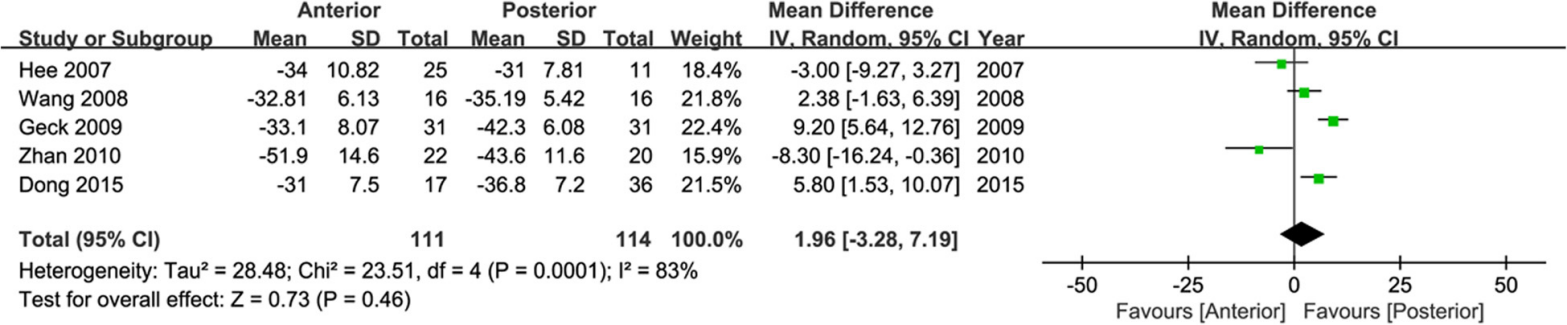
Forest plot for change values of TL/L curve

Data of TL/L curve were available in five articles [21, 22, 24, 25, 27]. No significant difference was showed in the change values of TL/L curve between the anterior and posterior approach (95 % CI −3.28 to 7.19, *P* = 0.46; *I*^2^ = 83 %; Fig. 3), from preoperation to final follow-up.

### Correction rate of TL/L curve

**Fig. 4:**
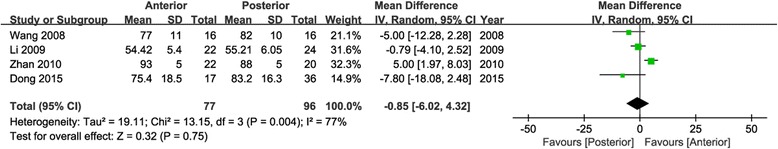
Forest plot for correction rate of TL/L curve

Data of TL/L curve correction rate were available in four articles [22, 23, 25, 27]. No significant difference was showed in TL/L curve correction rate of final follow-up between the two approaches (95 % CI −6.02 to 4.32, *P* = 0.75; *I*^2^ = 77 %; Fig. 4).

### Change values of TK

**Fig. 5:**
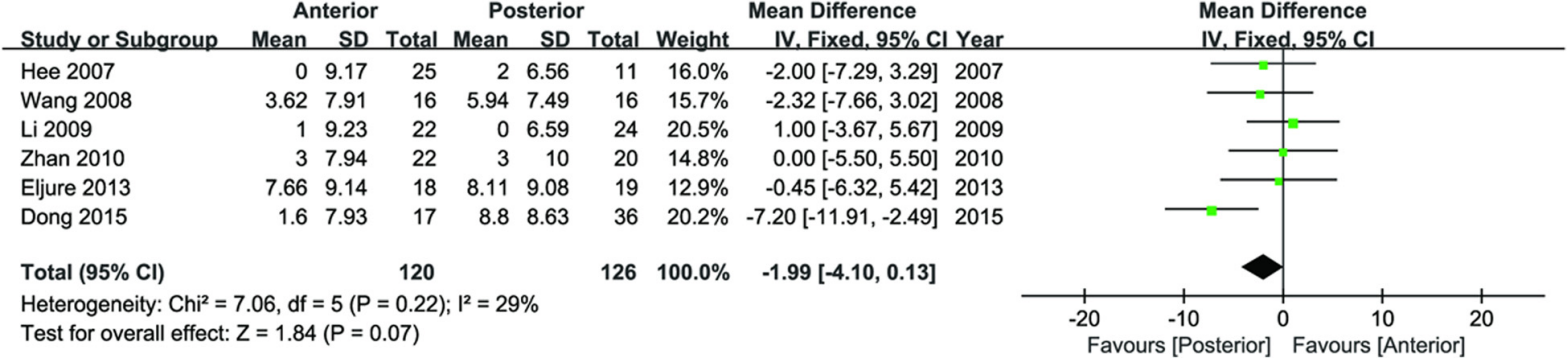
Forest plot for change values of TK

In the analysis of TK, data were extracted from six studies [21–23, 25–27]. No significant differences were found in the change values of TK between the anterior and posterior approach (95 % CI −4.10 to 0.13, *P* = 0.07; *I*^2^ = 29 %; Fig. 5), from preoperation to final follow-up.

### Change values for LL

**Fig. 6:**
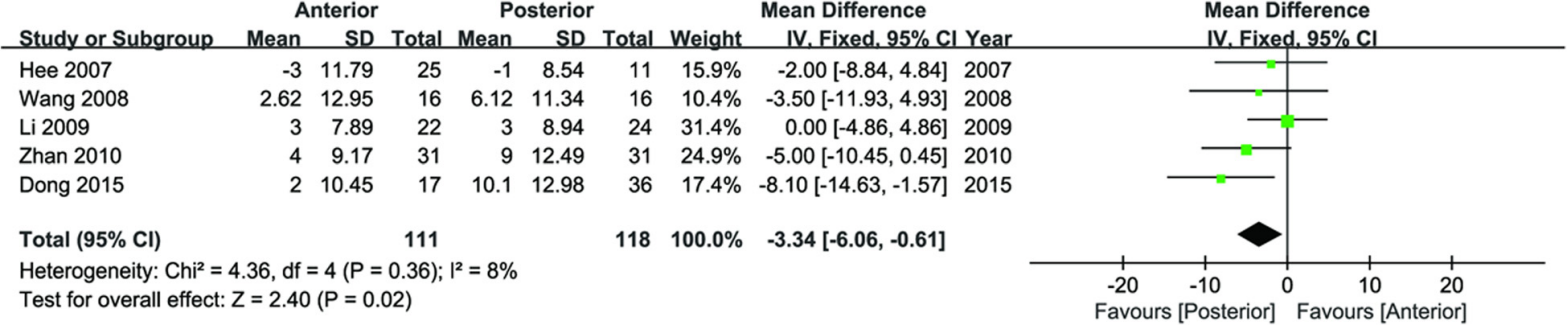
Forest plot for change values of LL

Data of LL were available in six articles [21–23, 25, 27]. According to the statistical analysis, significant difference was showed in the change values of LL (95 % CI −6.06 to −0.61, *P* = 0.02; *I*^2^ = 8 %; Fig. 6). The posterior approach obtained a larger increasing Cobb angle of LL than the anterior approach, from preoperation to final follow-up.

### Incidence of PJK

**Fig. 7:**
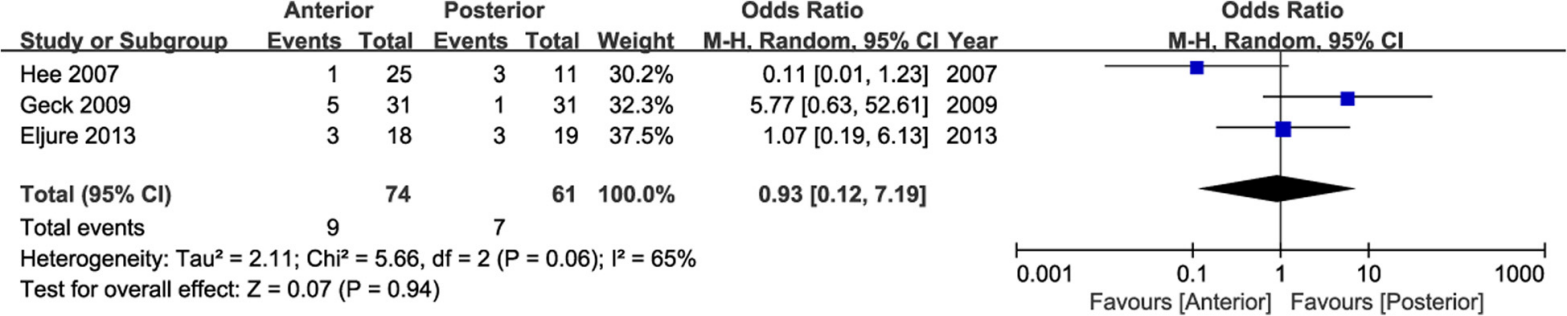
Forest plot for incidence of PJK

The data were extracted from three studies [21, 24, 26]. However, no significant difference was noted between the two approaches in incidence of PJK of final follow-up (95 % CI 0.12 to 7.19, *P* = 0.94; *I*^2^ = 65 %; Fig. 7).

## Discussion

The clinical outcome and early disc degeneration of saving one more fusion segment were still unclear. Helenius et al. reviewed 98 consecutive patients at a minimum of 19.1 years follow-up and concluded that no association between the spondylodesis fusion segment and the Scoliosis Research Society total score [30]. Danielsson et al. compared patients fused to L3 (*n* = 102) versus L4 (*n* = 37) and reported that saving one more segment had no clinical relevance at least 23 years follow-up [31]. Regarding to the early disc degeneration, Sudo et al. reported that patients with anterior approach did not preserve the patients from disc degeneration [32]. Perez-Grueso et al. reported a similar paper with a minimum follow-up of 10 years and concluded that patients who underwent spinal fusion surgery had a similar disc degeneration to general population [33].

Data of the fusion segments were extracted from seven studies. Compared with posterior approach, anterior approach represented a significant shorter fusion segments (95 % CI −1.72 to −0.71, *P* < 0.00001), which suggested that the anterior approach saved approximate one fusion segment. Although the clinical outcome and early disc degeneration of saving one fusion segment were still unclear, saving fusion segment indicates less anchors and less implant cost. Wang et al. reported the comparison of implant cost between the anterior and posterior approach ($ 6157 vs. 10,336, *P* < 0.0001) [12]. Medical expense was important to the decision of surgeons, especially in developing countries. Sudo et al. reported that the anterior approach produced satisfactory radiographic, clinical, and functional outcomes in Lenke 5C AIS with a mean of 17.2 years follow-up [32]. Therefore, the one less fusion segment of anterior approach was the potential advantage in Lenke 5C AIS.

The advantages of the anterior approach in Lenke 5C AIS had been recorded by many scholars. Verma et al. reported that anterior instrumentation for TL/L curves of AIS obtained optimal radiographic and clinical outcomes, relatively short fusion segments [34]. Maurice et al. considered that anterior approach in lumbar curves of AIS offered extremely acceptable radiological and surgical outcomes at mid-term follow-up of 6 years [35]. Furthermore, after long-term follow-up of 17 years, Kelly et al. reported that anterior approach offered satisfactory score of the SRS and Oswestry tests, excellent functional outcomes [36]. Sudo et al. reported that the anterior approach in Lenke 5C curves offered appropriate clinical measures and radiographic outcomes with acceptable impairment of pulmonary function, after a maximum follow-up of 23 years [37].

Posterior pedicle screw system was the most prevalent operating treatment in Lenke 5C curves. A large series of 114 TL/L curves was enrolled in the research, and it concluded that the average curve correction of posterior pedicle screw system was 66 % [38]. Bennett et al. conducted a study with TL/L patients, who were taken posterior spinal fusion with 5 years follow-up, and drew a conclusion that the curve correction of coronal plane and sagittal plane was well maintained [39]. Furthermore, the finite element analysis was used to estimate possible surgical results of the anterior and posterior approach in Lenke 5 curves, and Zhang et al. found that the posterior spinal fusion was regarded as the ideal surgical procedure [40].

In this study, data of TL/L curve were available in five articles, and no significant difference was observed in the change values of TL/L curve between anterior and posterior approach (95 % CI −3.28 to 7.19, *P* = 0.46). Similarly, no significant difference was showed in TL/L curve correction rate of final follow-up between the two approaches (95 % CI −6.02 to 4.32, *P* = 0.75).

Sagittal correction attracted more and more surgeons’ attention. Previous studies compared the anterior and posterior approach for sagittal plane and still did not come to an agreement [41]. TK was the critical parameter of sagittal correction. Sucato et al. reviewed multicenter surgical database of AIS and reported that the anterior approach (*n* = 135) remained greater TK than posterior instrumentation [18]. Schmidt et al. included 42 thoracic lordoscoliosis patients and concluded that anterior invasive technique obtained significantly better restoration of TK than posterior spinal fusion [42]. Izatt et al. found that TK was restored at mean Cobb angle of 11.8°, 2 years after anterior approach surgery [43], while Rushton et al. prospectively compared 42 consecutive patients and reported anterior invasive technique had no overall effect of sagittal correction while posterior spinal fusion significantly reduced kyphosis [44]. In the analysis of TK, data were extracted from six studies and no significant differences were found in the change values of TK between the anterior and posterior approach (95 % CI −4.10 to 0.13, *P* = 0.07), from preoperation to final follow-up.

PJK was a frequent cause of reoperation, and it could be defined as pathological kyphosis deformity adjacent from the caudal endplate of the upper instrumented vertebrae to the cephalad endplate of two vertebrae proximal and might be involved with retention of proximal intervertebral elements [45]. Mendoza-Lattes et al. found that sagittal balance and TK were the predictors of PJK according to logistic regression [46]. The data of PJK were extracted from three studies. However, no significant difference was noted between the two approaches in incidence of PJK of final follow-up (95 % CI 0.12 to 7.19, *P* = 0.94). LL was an important component in keeping sagittal balance. Data of LL were available in six articles, and significant difference was showed in the change values of LL (95 % CI −6.06 to −0.61, *P* = 0.02). The posterior approach obtained a larger increasing Cobb angle of LL than the anterior approach, from preoperation to final follow-up.

Some limitations should not be ignored in this meta-analysis. First, some important comparisons to evaluate the surgical treatment of AIS were limited. Such as hospital charges, quality of life, complications, and surgical revision rates, which issues were needed to be clarified in the future studies. Second, the sample size was not large and restricted by the low rates of Lenke 5C AIS. Third, most included articles were retrospective instead of RCT studies, and the quality of included articles was lower.

## Conclusions

This is the first meta-analysis comparing the two surgical approaches in Lenke 5C curves.

The anterior and posterior approach can obtain comparable coronal correction, change values of thoracic kyphosis, and incidence of proximal junctional kyphosis. The anterior approach saves approximate one more fusion segment, and the posterior approach can obtain a larger increasing Cobb angle of lumbar lordosis, from preoperation to final follow-up.

## Abbreviations

AIS, adolescent idiopathic scoliosis; LL, lumbar lordosis; NOS, Newcastle-Ottawa scale; PJK, proximal junctional kyphosis; RCT, randomized controlled trial; TK, thoracic kyphosis; TL/L, thoracolumbar/lumbar
